# LncRNA DNAJC3-AS1 Regulates Fatty Acid Synthase *via* the EGFR Pathway to Promote the Progression of Colorectal Cancer

**DOI:** 10.3389/fonc.2020.604534

**Published:** 2021-02-02

**Authors:** Yanyan Tang, Rui Tang, Mengtian Tang, Ping Huang, Zhiqiang Liao, Jumei Zhou, Lianqing Zhou, Min Su, Pan Chen, Jiarui Jiang, Yingbin Hu, Yujuan Zhou, QianJin Liao, Zhaoyang Zeng, Wei Xiong, Junhong Chen, Shaolin Nie

**Affiliations:** ^1^ Hunan Cancer Hospital and The Affiliated Cancer Hospital of Xiangya School of Medicine, Central South University, Changsha, China; ^2^ Department of Colorectal Surgery, Hunan Cancer Hospital and the Affiliated Cancer Hospital of Xiangya School of Medicine, Central South University, Changsha, China; ^3^ Central Laboratory, The Affiliated Cancer Hospital of Xiangya Medical School, Central South University, Changsha, China; ^4^ Department of Ultrasound, Department of Stomatology, Third Xiangya Hospital, Central South University, Changsha, China; ^5^ The University of South China, Hengyang, China; ^6^ The Key Laboratory of Carcinogenesis and Cancer Invasion of the Chinese Ministry of Education, Cancer Research Institute, Central South University, Changsha, China

**Keywords:** long non-coding RNA, DNAJC3-AS1, epidermal growth factor receptor pathway, fatty acid synthase, colorectal cancer

## Abstract

Colorectal cancer (CRC) is one of the most common cancers worldwide. Recent studies have shown that long non-coding RNAs (lncRNAs) are involved in tumorigenesis and the development of CRC. By constructing a differential lncRNA expression profile, we screened gene chips and found that DNAJC3-AS1 was highly expressed in CRC tissues and was associated with poor prognosis in patients with CRC. Further, we proved through assays such as wound healing, colony formation, and Cell Counting Kit-8 (CCK8) that interfering with DNAJC3-AS1 could reduce the proliferation, migration, and invasion of CRC cells. Mechanically, we found that DNAJC3-AS1 regulates fatty acid synthase to promote the progression of CRC *via* the epidermal growth factor receptor/phosphatidylinositol 3-kinase/protein kinase B/nuclear factor κB signaling pathway. Therefore, DNAJC3-AS1 may be a new target for the diagnosis and therapy of CRC.

## Introduction

Colorectal cancer (CRC) is the most common gastrointestinal cancer worldwide and has a high morbidity and mortality rate (1–3) and the incidence of CRC has been associated with poor eating habits, with the number of cases increasing annually (3, 4). Abnormal lipid metabolism, such as that in case of obesity, is a major risk factor that promotes tumorigenesis and the development of CRC. Obesity can induce metabolic changes in colon cells and induce oncogene expression with age (5).

Lipid metabolism reprogramming has emerged as a new hallmark of cancer (6, 7). Early tumor growth requires nutrient absorption and biosynthesis (8). In rapidly proliferating tumor cells, the *de novo* synthesis of fatty acid is enhanced and the expression of key fatty acid synthesis enzymes, including acetyl CoA carboxylase (ACC) and fatty acid synthase (FASN), is increased. After the tumor cells synthesise fatty acids, they further synthesise triglycerides or sterol esters and then store them in lipid droplets. The accumulated lipids can protect tumor cells from harsh environments and continuously provide tumor cells with phospholipids for developing the membrane structure. In addition, these tumor metabolites can act as signal molecules in signaling pathways and contribute to local invasion and metastasis of tumor cells. In the later stages of tumor progression, drug resistance also causes the development of new metabolic phenotypes. Studies have shown that fibroblasts in the tumor microenvironment highly express FASN, which mediates lipid metabolism reprogramming, causes the accumulation of several fatty acids, and promotes the migration of CRC cells (9). However, the regulatory mechanisms of lipid metabolism reprogramming in CRC are not fully understood.

Long non-coding RNAs (lncRNAs) are a group of RNA transcripts that span more than 200 nucleotides in length and have limited protein-coding ability (10). Several studies have shown that lncRNAs can directly act as tumor suppressors or oncogenes or play a role in the regulation of tumor suppressors or oncogenes at the transcriptional or post-transcriptional levels (11). Many studies have confirmed that lncRNAs play an important role in the occurrence and development of CRC (12–15). Recently, a concern has been raised that lncRNAs regulate cancer progression *via* participating in the lipid metabolism. For example, LNMICC promotes cervical cancer metastasis *via* reprogramming fatty acid metabolism. Furthermore, TINCR promotes nasopharyngeal carcinoma (NPC) proliferation and metastasis by upregulating acetyl-CoA levels, and ultimately promoting lipid biosynthesis (16).

It is reported that EGFR is overexpressed in many types of cancers, and activates various downstream signaling pathways including the phosphatidylinositol 3-kinase (PI3K)/Akt pathway (17). The epidermal growth factor receptor (EGFR)/PI3K/Akt/nuclear factor κB (NF-κB) signaling pathway plays an important role in the occurrence and development of tumors, especially in the proliferation, invasion, and metastasis of tumor cells (18–21). Moreover, EGFR/PI3K/Akt has been demonstrated to activate SREBP1 cleavage and up-regulates ACC1 and FASN, leading to enhanced lipid metabolism (22, 23). The accumulate evidences inspired us to explore the role of lncRNAs in the link between lipid metabolism and CRC.

In the present study, we aimed to profile the expression patterns and dysregulation of lncRNAs in CRC by analysing Gene Expression Omnibus (GEO) data sets (GSE21510, GSE22598, GSE23878, GSE32323, and GES39582). We focused on the lncRNA DNAJC3-AS1, the expression of which was markedly overexpressed and was correlated with BMI, poor patient prognosis. Further study demonstrated that DNAJC3-AS1 could promote CRC proliferation, metastasis, invasion, and lipid accumulation. In addition, we found that DNAJC3-AS1 regulated the EGFR/PI3K/AKT/NF-κB/SREBP signaling pathway and correlated with the expression of key fatty acid synthesis enzymes, including ACC1 and FASN.

## Materials and Methods

### Data Set Analysis

The original CRC gene expression profile data and related clinical data of six independent gene chips GSE21510, GSE22598, GSE23878, GSE32323, and GES39582 were obtained from the GEO database. GSE21510 contains 104 tumor and 44 normal tissues, GSE22598 contains 17 tumor and 17 normal tissues, GSE23878 contains 35 tumor and 24 normal tissues, GSE32323 contains 17 tumor and 17 normal tissues, and GES39582 contains 233 tumor and 19 normal tissues. Significant Analysis of Microarray software was used to analyze the expression differences of lncRNAs in normal colorectal mucosal and CRC tissue samples from the two groups (**Supplementary Table 2**).

### Clinical Samples

Tumor tissues paired with normal colorectal mucosal tissues were obtained from 36 patients with CRC diagnosed and operated on at the Hunan Cancer Hospital from 2017 to 2019. After collection, the tissue specimens were immediately frozen in liquid nitrogen. In addition, *in situ* hybridization assays were performed on 53 patients who underwent surgery at the Human Provincial Cancer Hospital from 2010 to 2013. 10 normal samples were obtained from adjacent tissues. All specimens were obtained from surgically removed tissue specimens from patients with preoperatively confirmed CRC who had not received radiation or chemotherapy. This study was approved by the ethics committee of the Hunan Cancer Hospital, and written informed consent was obtained from all patients.

### *In Situ* Hybridization (ISH)

Tissue samples from 53 patients with CRC were fixed with 4% paraformaldehyde, embedded in paraffin, and sliced. The expression of DNAJC3-AS1 in CRC tissues was measured with an oligonucleotide probe. The following oligonucleotide probes were purchased from BOSTER Company (Wuhan, China): 5′-TGAAT TATAA ATAGC ATAGT GAATT TGTGA TTCCC TGAAG-3′, 5′-TCCCT CCTTC CTTGA GTGTG GGCTG GACTT AGTGA CTAAC-3′, 5′-TTGGG ACCTC CCTGT ATTAT CCTTA TGCCC TTACT TGAAA-3′, and 5′-labeled with a digoxigenin-deoxyuridine triphosphate tag. The experiment was conducted according to the manufacturer’s protocol using a sensitive, enhanced ISH kit (Boster, Wuhan, China). A semi-quantitative scoring standard was used to record the staining intensity and positive area of the slice. The scoring was graded as 0 (negative), 1 (<10% positive), 2 (10%–50% positive), or 3 (>50% positive) in accordance with the staining proportion and intensity. The final scores were regarded as low expression (0–1) and high expression (2–3). All sections were evaluated blindly by two pathologists.

### *In Situ* Hybridization Evaluation Criteria

Two pathologists scored the results according to the following criteria: (1) staining intensity: no observed cell staining was scored as 0; cells with light-brown cell staining as 1; cells stained brown with no background staining, or dark-brown stained cells with light-brown background staining were recorded as moderately positive, as 2; dark-brown stained cells with no background staining were recorded as strongly positive, as 3. (2) number of positive cells: no positive cells were scored as 0; less than 25% of positive cells as 1; between 25 and 50% positive cells as 2; positive cells over 50% recorded as strongly positive, as 3. The final scores were obtained by multiplying the two scores. The results were as follows: 0 was considered as negative expression, and the final score was 0; 1−2, as weakly positive, and the final score was 1; 3 to 4, as moderately positive, and the final score was 2; 6 to 9 as strong positive, and the final score was 3.

All the target cells of each tissue were counted under 10× magnification, and the counting was repeated twice. The average values obtained from the counting was used for statistical analysis. ISH scores of 1, 2, and 3 indicated high expression of DNAJC3-AS1, and IHC scores of 0 indicated low expression of DNAJC3-AS1. All the samples were scored independently by two experienced pathologists who were double-blinded.

### RNA Isolation and Quantitative Reverse Transcription-Polymerase Chain Reaction (qRT-PCR)

Total RNA was extracted using TRIzol reagent according to the manufacturer’s protocol (Invitrogen, USA). cDNA was synthesized using a reverse transcription kit (Bio-Rad, Hercules, CA, USA). qRT-PCR was performed using SYBR Green (Bio-rad, Hercules, CA, USA) with a LightCycler 480 RT-PCR detection system (Roche). β-actin was used as an internal control for normalization. The expression of DNAJC3-AS1 was normalized to the corresponding β-actin expression level. The following equation was used to calculate the relative expression: ΔCt = Ct (target gene) − Ct (β-actin), fold expression = 2 – [ΔCt (tumor) − ΔCt (normal)] divided by the Cq value. The primer sequences used in the experiment were as follows: DNAJC3-AS1: 5′-AGCGATTGTGGAAGACCCTG-3′, 5′-ATTTCCCCTGGTAAGCGCAA-3′ and β-actin: 5′-TCACCAACTGGGACGACATG-3′, 5′-GTCACCGGAGTCCATCACGAT-3′.

### Cell Lines and Cell Transfection

The CRC cell lines HT29, HCT116, SW480, and SW620 and normal human colon tissue cells CCD-18Co were obtained from the Cancer Institute of Xiangya Medical College, Central South University. The CRC cell lines HT29 and SW620 were cultured in RPMI-1640 medium (BI) with 10% foetal bovine serum (FBS, ZETA) and maintained at 5% CO_2_ and 37°C. The colorectal cell line CCD-18CO and the CRC cell lines HCT116 and SW480 were cultured in DMEM (Gibco, Beijing, China) with 10% FBS (ZETA).

A small interfering RNA (siRNA) was used to interfere with the expression of DNAJC3-AS1 in cells. Cells were seeded in a 12-well plate in Opti-MEM medium (Invitrogen) and transfected with an siRNA targeting DNAJC3-AS1 or a scrambled control siRNA using Hiperfect (Takara). The sequences of the siRNA targeting DNAJC3-AS1 were as follows: siDNAJC3-AS1: sense 5′- GGAAGCACAGUCUCAACUUTT-3′ and antisense: 5′- AAGUUGAGACUGUGCUUCCTT-3′. The sequences of the scrambled control siRNA were provided by Sangon Biotech Company (Shanghai, China).

### Cell Counting Kit (CCK)-8 and Colony Formation Assays

The proliferation ability of the cells was evaluated by CCK-8 and colony formation assays. In the CCK-8 assay, 100 µl of suspension containing 1 × 10^3^ CRC cells was inoculated into each 96-well plate. Ten microliters of CCK-8 solution was added to each well, and the cells were incubated at 37°C for 4 h. The absorbance at 450 nm was measured at the indicated time points (24, 48, 72, and 96 h after transfection).

In the colony formation assay, 2 × 10^3^ CRC cells per well were seeded in a 6-well plate, cultured in 2 mL DMEM containing 10% FBS and 2 mL of RPMI 1640 medium containing 10% FBS, and incubated at 37°C and 5% CO_2_ for 24 h. The medium was changed every 2 days, and the culture was terminated on the 12^th^ day. The cells were immobilized with 4% paraformaldehyde for 30 min, purified twice with phosphate-buffered saline (PBS), and stained with 2.5% crystal violet. Subsequently, live colonies were counted.

### Cell Migration and Invasion Assays

The migration ability of CRC cells was measured by wound healing assays. The cells were seeded in a 6-well plate and grown until 70% confluence. Vertical scratches in the cell monolayer were created using a 10-µl pipette tip, and the cells were washed three times with PBS to remove cell debris. The width of the wound was measured under a microscope at 0, 24, and 48 h after the scratch wound formation, and the wound area was calculated using Image J.

The invasion ability of CRC cells was evaluated by the Matrigel Transwell invasion assay. A total of 2 × 10^5^ cells in 200 µl serum-free medium were added to the top of a Matrigel-coated Transwell cell culture chamber (8-μm pore size, BD Biosciences, New Jersey, USA), and cells in 600 µl of medium containing 20% FBS were added to the bottom of the chamber. Cells were then incubated at 37°C for 24 h. The cells that migrated or invaded the lower cavity were fixed with 4% paraformaldehyde and stained with 2.5% crystal violet. The cells on the upper surface of the chamber were gently wiped with a cotton swab. The number of invasive cells was counted from six randomly selected 100× fields under a microscope and displayed as the average value of each field.

### Nile Red Staining

To visualize lipid droplets, cultured cells were fixed in 4% paraformaldehyde solution on the 6-well plates, stained with 0.05 μg/ml Nile red (Solarbio) for 10 min, washed with PBS, then stained with DAPI. The images were visualized by immunofluorescence microscopy.

### Western Blotting

Total protein was extracted from the cells using RIPA buffer (Boster, China) containing a phosphatase inhibitor and quantified using the bicinchoninic acid protein detection kit (Invitrogen, USA) according to the manufacturer’s instructions. After denaturation, 20 mg of protein samples were loaded into a sodium dodecyl sulfate-polyacrylamide gel electrophoresis gel for electrophoresis. After transfer of protein bands, the polyvinylidene fluoride membrane (Invitrogen, USA) was blocked with bovine serum albumin (Invitrogen, USA) at 25°C for 1 h and incubated with the following primary antibodies overnight at 4°C: anti-phospho-EGFR (1:1,000, Abclone), anti-phosphorylated PI3K (1:1,000, Abclone), anti-phosphorylated AKT (1:1,000, Abclone), anti-phosphorylated NF-κB (1:1,000, Abclone), anti-SREBP1 (1:1,000, Abcam), anti-ACC1 (1:1000, Proteintech), anti-FASN (1:1,000, Proteintech), and β-actin (1:1,000, Abclone). After washing with Tris-buffered saline with 0.1% Tween^®^ 20 (TBS-T), the membrane was incubated with an anti-rabbit secondary antibody for 2 h at room temperature. After another washing with TBS-T, protein bands were visualized using an enhanced chemiluminescent substrate kit (Obsen, Beijing). Experiments were performed in triplicate.

### Statistical Analysis

All assays were performed independently at least three times. All statistical analyses were performed using Microsoft Excel 2007 version (Microsoft, USA) and GraphPad Prism version 5.0 (GraphPad Software Inc., San Diego, CA, USA) software. Data are expressed as the mean ± standard error of the mean. Differences between two independent groups were assessed by Student’s t-test, and the differences in multiple comparisons were assessed by one-way analysis of variance. Kaplan–Meier analysis was used to plot survival curves, and log-rank test was used for analysis. Comparisons among categorical variables were performed using χ^2^ or Fisher’s exact tests. A two-tailed P-value of 0.05 or less was considered statistically significant.

## Results

### DNAJC3-AS1 Is Upregulated in CRC and Predicts Poor Prognosis

**Figure 1:**
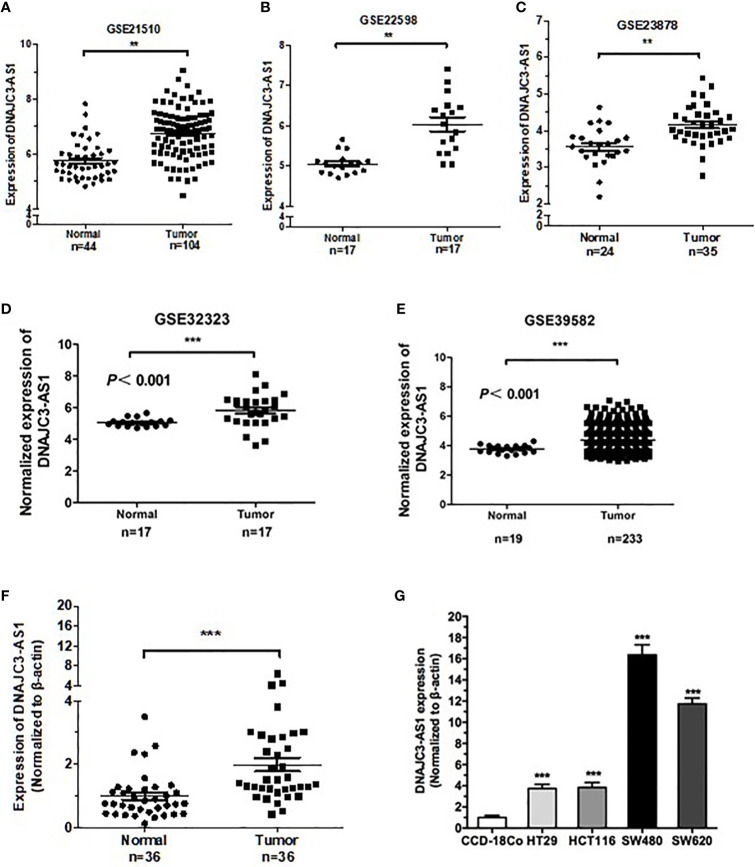
DNAJC3-AS1 is highly expressed in CRC. **(A–E)** DNAJC3-AS1 expression, obtained from the Gene Expression Omnibus (GEO) database, is upregulated in CRC tissues compared with that in normal colorectal tissues in GSE21510, GSE22598, GSE23878, GSE32323, and GES39582 datasets. **(F)** DNAJC3-AS1 expression is higher in CRC tissue samples (n = 36) than in adjacent normal tissues (n = 36). **(G)** DNAJC3-AS1 expression is significantly higher in the CRC cell lines HT29, HCT116, SW480, and SW620 than in the normal colon cell line CCD-18Co. (**P < 0.01 and ***P < 0.001).

Reanalysis of the GEO datasets (GSE21510, GSE22598, GSE23878, GSE32323, and GES39582) showed that DNAJC3-AS1 was significantly upregulated in CRC tissues compared with that in non-tumor tissues (**Figures 1A–E**).

To verify the results of the datasets, we collected 36 CRC tissues paired with normal tissues and detected the expression of DNAJC3-AS1 by qRT-PCR. The expression of DNAJC3-AS1 in CRC tissues was significantly increased compared with that in the corresponding normal tissues (**Figure 1F**). In addition, DNAJC3-AS1 was significantly upregulated in the four CRC cell lines HT29, HCT116, SW480, and SW620 compared with that in the normal colon cell line CCD-18Co (**Figure 1G**).

**Figure 2:**
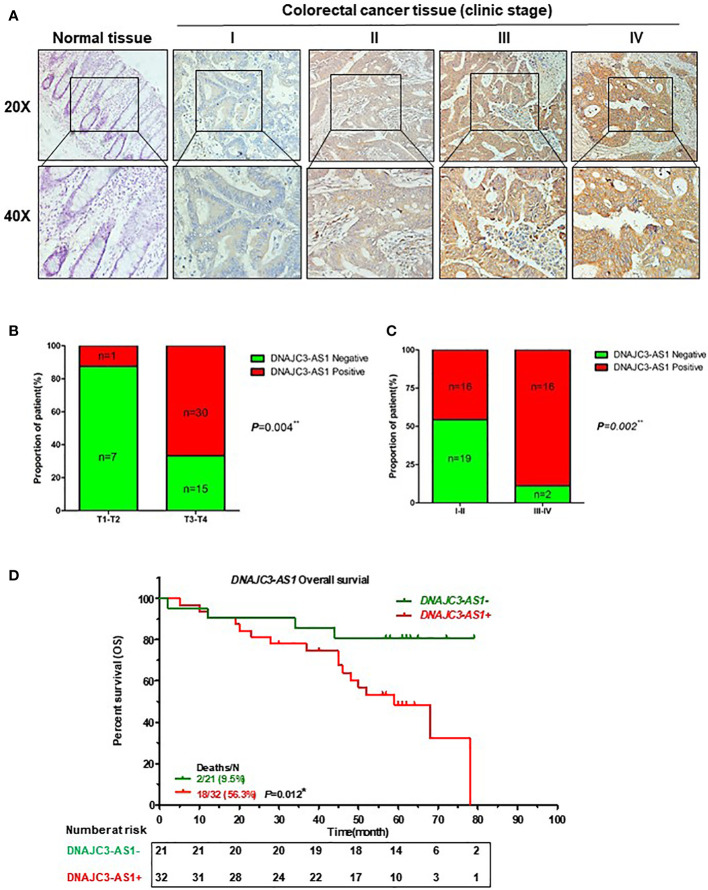
High expression of DNAJC3-AS1 is associated with poor prognosis of patients with CRC. **(A)** DNAJC3-AS1 expression measured by *in situ* hybridization in paraffin-embedded CRC biopsy samples. Upper panel: magnification = 20×; lower panel: magnification = 40×. **(B, C)** DNAJC3-AS1 expression is associated with TNM staging. **(D)** Highly expression of DNAJC3-AS1 is correlated with shorter overall survival. (*P < 0.05, **P < 0.01).

We further verified the expression of DNAJC3-AS1 in CRC tissues and its correlation with clinicpathological parameters of patients with CRC using ISH. DNAJC3-AS1 was highly expressed in the cancer nests of colorectal cancer tissues compared with that in the adjacent normal colorectal tissues (**Figure 2A**). We analyzed the relationship between DNAJC3-AS1 expression and clinicopathological parameters, such as the level of local invasion (T stage), lymphatic invasion (N stage), and BMI. The data showed that DNAJC3-AS1 expression was positively correlated with local invasion (T stage), I–IV stage, and BMI (**Figures 2B, C**, **Supplementary Table S1**). High expression of DNAJC3-AS1 was also associated with shortened overall survival of patients with CRC, suggesting that upregulation of DNAJC3-AS1 is a predictor of poor prognosis (**Figure 2D**). The above results indicated that the high expression of DNAJC3-AS1 in CRC is associated with higher local infiltration, TNM stage, BMI, and poor prognosis.

### DNAJC3-AS1 Can Promote the Proliferation of CRC Cells

**Figure 3:**
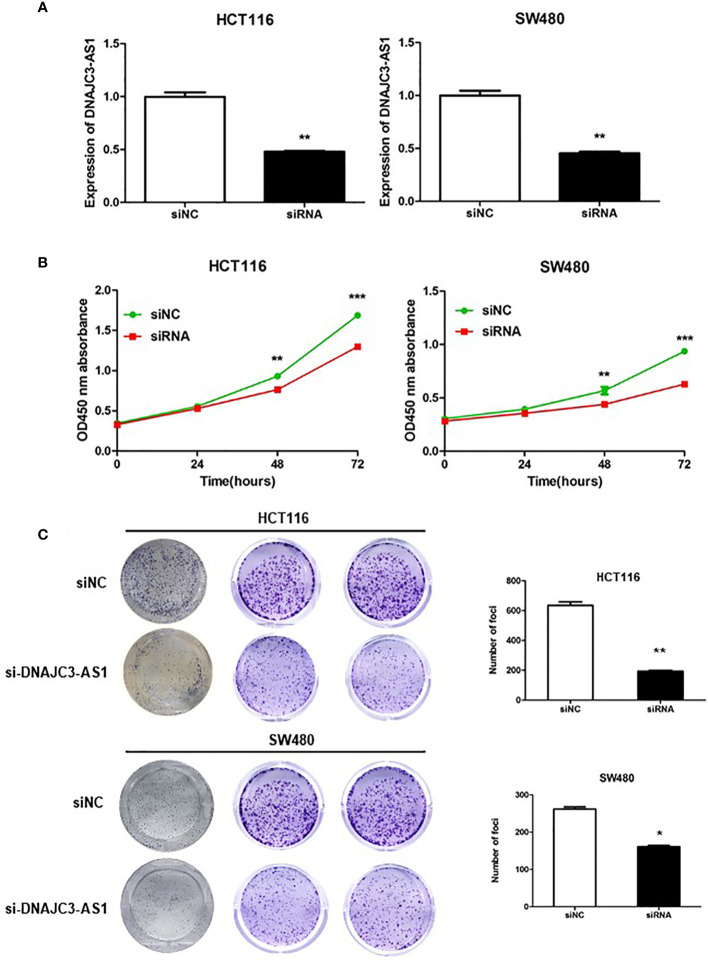
DNAJC3-AS1 can promote the proliferation of CRC cells. **(A)** Following siRNA transfection, the expression of DNAJC3-AS1 in HCT116 and SW480 cells is significantly inhibited (P = 0.007, P = 0.003). **(B)** CCK-8 assay shows that knockdown of DNAJC3-AS1 significantly inhibits the proliferation of HCT116 and SW480 cells (P < 0.001, P < 0.001). **(C)** Colony formation assay shows that knockdown of DNAJC3-AS1 significantly reduces the number of cancer cell colonies (*P < 0.05, **P < 0.01 and ***P < 0.001).

To further analyze the biological function of DNAJC3-AS1 in CRC, HCT116 and SW480 cells were transfected with siRNA to knock down the expression of DNAC3 AS1 in CRC cells. The transfection effect was detected by qRT-PCR. The results showed that after siRNA transfection, the expression of DNAJC3-AS1 in HCT116 and SW480 cells was significantly inhibited (**Figure 3A**). CCK-8 assays revealed that knockdown of DNAJC3-AS1 significantly inhibited the proliferation of HCT116 and SW480 cells (**Figure 3B**). Results of the colony formation assay were consistent with those of the CCK-8 assay; the knockdown of DNAJC3-AS1 significantly reduced the number of clone colonies (**Figure 3C**).

### DNAJC3-AS1 Can Promote the Migration and Invasion and Lipid Accumulation of CRC Cells

**Figure 4:**
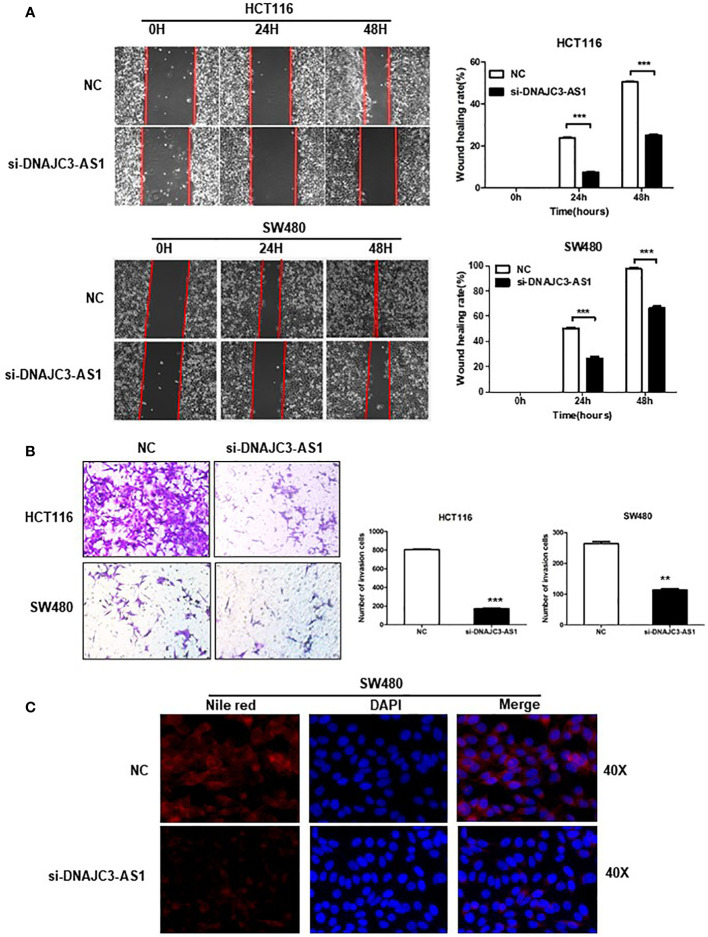
DNAJC3-AS1 can promote the migration and invasion of CRC cells. **(A)** Wound healing assay shows that knockdown of DNAJC3-AS1 can significantly reduce the migration ability of HCT116 and SW480 cells (P < 0.001, P < 0.001). **(B)** Transwell assays show that knockdown of DNAJC3-AS1 significantly inhibits the invasion ability of HCT116 and SW480 cells. **(C)** Cellular neutral lipids were measured in SW480 cells expressing NC, si-DNAJC3-AS1 by Nile red staining. magnification = 40×. (**P < 0.01 and ***P < 0.001).

We performed a wound-healing assay to analyze the influence of DNAJC3-AS1 on the migration ability of CRC cells. The results showed that knockdown of DNAJC3-AS1 can significantly reduce the migration ability of HCT116 and SW480 cells (**Figure 4A**). Finally, we used the Transwell assays to analyze the effect of DNAJC3-AS1 on the invasion ability of CRC cells. The results showed that knockdown of DNAJC3-AS1 significantly inhibited the invasion ability of HCT116 and SW480 cells (**Figure 4B**). Therefore, the above results confirmed that DNAJC3-AS1 knockdown was effective in CRC cells and could inhibit the migration and invasion abilities of CRC cells. Therefore, the high expression of DNAJC3-AS1 serves as an oncogene in CRC.

Based on our previous finding that the higher DNAJC3-AS1expression level correlated with BMI (*P*=0.038) (**Supplementary Table S1**). We further evaluate the effects of DNAJC3-AS1 on lipid content in CRC cell lines SW480. Our data demonstrated that DNAJC3-AS1 knockdown induced decreased levels of lipid accumulation (Nile red staining) in SW480 (**Figure 4C**).

### DNAJC3-AS1 Regulates the Expression of ACC1/FASN Via EGFR/PI3K/AKT/NF-Kb/SREBP1 Signaling Pathway in CRC

**Figure 5:**
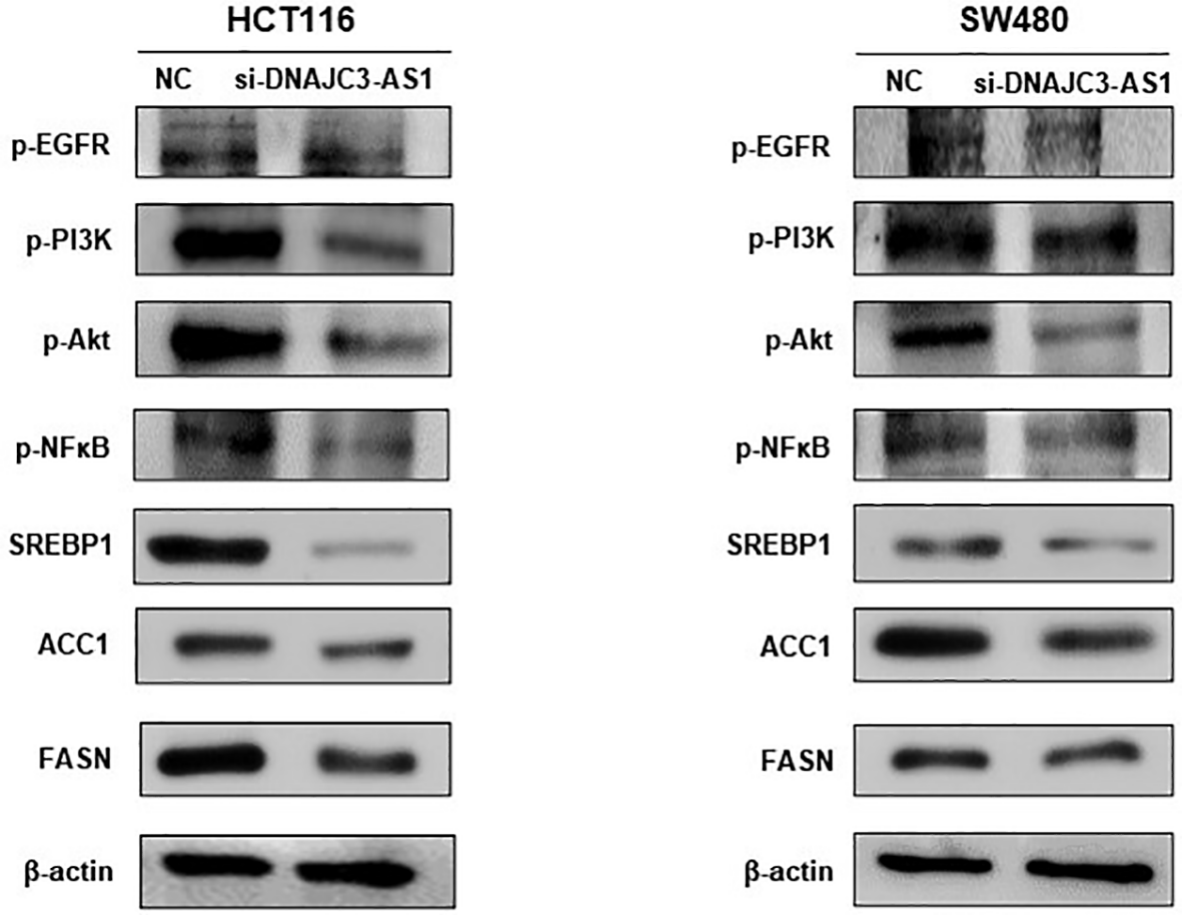
DNAJC3-AS1 regulates the expression of fatty acid synthase *via* EGFR/PI3K/AKT/NF-κB signaling pathway. Expression of EGFR/PI3K/AKT/NF-κB pathway-associated proteins and their corresponding phosphorylated levels are detected by western blotting in HCT116 and SW480 cells transfected with NC or DNAJC3-AS1 siRNA. β-actin is used as an internal control.

To provide further evidence, we measured the expression of FFA metabolic enzymes and related signaling pathway. In our study, western blotting showed that knockdown of DNAJC3-AS1 significantly reduced the expression levels of P-EGFR, P-PI3K, P-Akt, P-NF-κB, and SREBP1 in HCT116 and SW480 cells (**Figure 5**). The EGFR/PI3K/AKT/sterol regulatory element-binding protein (SREBP1) signaling pathway is closely related to lipid metabolism. Through western blotting, we found that knockdown of the *DNAJC3-AS1* gene decreased the expression levels of ACC1 and FASN in HCT116 and SW480 cells (**Figure 5**). Therefore, DNAJC3-AS1 may regulate the expression of ACC1/FASN *via* the EGFR/PI3K/AKT/NF-Kb/SREBP1 pathway to promote the progression of CRC.

## Discussion

CRC is one of the most common cancers and the second leading cause of cancer-related deaths (3). Therefore, there is an urgent need to understand the mechanism of CRC progression. In recent years, an increasing number of studies have shown that lncRNAs are closely related to the reprogramming of lipid metabolism and the occurrence and development of malignant tumors (12, 24–27). In this study, we mainly discussed the function and mechanism of lncRNA DNAJC3-AS1 in CRC.

First, we selected lncRNAs that were differentially expressed in CRC from the GEO database and verified them using qRT-PCR to screen out lncRNA DNAJC3-AS1 that was significantly highly expressed in CRC tissues and cells. Moreover, ISH assays confirmed that the expression of DNAJC3-AS1 is associated with local infiltration of CRC, TNM staging, and poor prognosis of patients. These results suggest that the expression of DNAJC3-AS1 is closely related to the occurrence of CRC. Next, we found through *in vitro* experiments that knockdown of DNAJC3-AS1 can significantly inhibit the proliferation, migration, and invasion of CRC cells, confirming that DNAJC3-AS1 acts as an oncogene in the development of CRC. To our knowledge, this study is the first to reveal the role of DNAJC3-AS1 in promoting the growth and metastasis of CRC cells. Western blotting further showed that DNAJC3-AS1 is closely related to the EGFR/NF-κB pathway.

EGFR, a cell-surface transmembrane receptor of tyrosine kinases, is a member of the ErbB family and plays an important role in the progression of CRC (28, 29). The binding of EGF to its receptor EGFR can initiate autophosphorylation of intracellular domains through tyrosine kinase activity and activate multiple downstream signal transduction pathways, including the PI3K/AKT pathway. The PI3K/AKT signal transduction pathway is a widely recognized carcinogenic pathway that plays a key role in a variety of tumors, including lung cancer (30), gastric cancer (31) and liver cancer (32). Moreover, several studies have shown that the PI3K/AKT signaling pathway is activated in CRC and plays a crucial role in regulating cell proliferation and maintaining the biological characteristics of malignant cells (33, 34). PI3K can generate phosphatidylinositol 3,4,5-trisphosphate in the plasma membrane, which then interacts with the PH domain of AKT to cause AKT aggregation. Subsequently, the increase in AKT expression or activity is recognized as the first step in the progression of various types of tumors (35). In addition, the PI3K/AKT signaling pathway is stimulated by a variety of oncogenes and growth factor receptors, such as insulin receptor tyrosine kinase, insulin-like growth factor 1 receptor, EGFR, and platelet-derived growth factor receptor (36). The abnormal regulation of NF-κB and the signaling pathways that control its activity are associated with the development and maintenance of cancer (37). Generally, NF-κB activity is regulated by its interaction with the inhibitory IκB (the nuclear localization signal of a given NF-κB dimer that interferes with DNA binding). Phosphorylation activates the IκB kinase (IκK) complex, which degrades the IκB proteasome, thereby activating NF-κB. The activation of IκK induces the phosphorylation of IκB and facilitates its degradation by the proteasome. At the same time, NF-κB enters the nucleus and activates the corresponding target gene (38). Studies have shown that PI3K can regulate the expression of NF-κB through phosphorylation and activation of AKT, thereby affecting cell survival, proliferation, metastasis, and other biological processes (39).

Many studies have reported that the EGFR/NF-κB signaling pathway is closely related to lipid metabolism. Lipid metabolism changes, especially FA synthesis (FAS) and fatty acid oxidation, are considered to be important metabolic reprogramming phenomena in tumor cells (40). ACC is a rate-limiting enzyme that catalyses the FAS pathway. There are two main isoforms of this protein. Cytoplasmic ACC1 exists in adipogenic tissues, and ACC2 bound to the outer mitochondrial membrane exists in lipids (41). Studies have shown that the high expression of ACC1 is associated with vascular invasion and disease recurrence in patients with hepatocellular carcinoma (42). FASN is a multi-enzyme protein complex that catalyses the biosynthesis of saturated fatty acids. Several studies have shown that FASN is overexpressed in a variety of cancers, such as breast cancer, prostate cancer, ovarian cancer, and CRC (43, 44). Studies have shown that the EGFR/PI3K/AKT/SREBP-1 signaling pathway can promote the progression of glioblastoma (22).

Therefore, in this study, we observed that DNAJC3-AS1 plays a role in the activation of the PI3K/AKT signaling pathway and regulates the expression of FASN in CRC cells. The expression of DNAJC3-AS1 was positively correlated with the expression levels of p-EGFR, p-PI3K, p-AKT, p-NF-κB, ACC1, and FASN. Therefore, we believe that the role of lncRNA DNAJC3-AS1 in the occurrence of CRC is partly mediated by the activation of the EGFR/PI3K/AKT/NF-κB/ACC1 and FASN signaling pathways. These findings indicate that DNAJC3-AS1 can regulate FASN *via* the EGFR/PI3K/AKT/NF-κB pathway to promote the progression of CRC and thus can be a candidate for molecular targeted therapy for CRC.

## Data Availability Statement

The data sets presented in this study can be found in online repositories. The names of the repository/repositories and accession number(s) can be found in the article.

## Ethics Statement

This study was approved by the Ethics Committee of Hunan Cancer Hospital, and written informed consent was signed.

## Author Contributions

YT, RT, and MT wrote the manuscript and did the experiments. PH and ZL revised the manuscript. JZ, LZ, MS, PC, JJ, YH, YZ, QL, ZZ, and WX helped in the revision of the manuscript. JC and SN revised the manuscript. All authors contributed to the article and approved the submitted version.

## Funding

This work was supported by the National Natural Science Foundation of China (82002873, 81972636, 81872281, 81472595), Natural Science Foundation of Hunan Province (2018JJ3634, 2019JJ40175, 2019JJ40183, 2018JJ1013), Research Projects of Health Commission of Hunan Province (20201772, B2019082, B20180400, B20180582), Research Projects of Health Department of Hunan Province (B2013-096), Changsha Science and Technology Project (kq2004139, kq1901071, kq1901072, kq1706045, kq1706043), and Ascend Foundation of National Cancer Centre (NCC2018b68).

## Conflict of Interest

The authors declare that the research was conducted in the absence of any commercial or financial relationships that could be construed as a potential conflict of interest.
